# Nanomaterials in Plants: A Review of Hazard and Applications in the Agri-Food Sector

**DOI:** 10.3390/nano9081094

**Published:** 2019-07-30

**Authors:** Eva Kranjc, Damjana Drobne

**Affiliations:** Department of Biology, Biotechnical Faculty, University of Ljubljana, Večna pot 111, 1000 Ljubljana, Slovenia

**Keywords:** engineered nanomaterials, plant phytotoxicity, agriculture, nanotechnology, nanosafety

## Abstract

Agricultural food crop plants interact with engineered nanomaterials (ENMs) from the application of agri-food nanotechnologies and from unintentional emissions originating from other nanotechnologies. Both types of exposure present implications for agricultural yield and quality, food chain transfer, and environmental and human health. In this review, the most recent findings from agricultural plant-ENM studies published in 2017 and 2018 are summarized. The aim of this is to identify the current hazard potential of ENMs for plants grown under typical field conditions that originate from both intentional and unintentional exposures and to contribute to knowledge-based decisions on the application of ENMs in food-agriculture. We also address recent knowledge on ENM adsorption, internalization, translocation, and bioaccumulation by plants, ENM impacts on agricultural crop yield and nutrition, and ENM biotransformation. Using adverse effect level concentrations and data on ENM accumulation in environmental matrices, the literature analyses revealed that C-, Ag-, Ce-, and Ti-based ENMs are unlikely to pose a risk to plants grown under typical field conditions, whereas Cu- and Zn-based ENMs require surveillance. Since multiple factors (e.g., ENM concentration, route of exposure, and plant type) influence the effects of ENMs on plants, biomonitoring is recommended for tracking ENM environmental exposure in the future.

## 1. Introduction

The 25th meeting of the Working Group for the Safety of Novel Foods and Feeds (WG-SNFF) in June 2018 recognized that the potential risks and benefits of nanotechnology-based products are examined on a case-by-case approach, as it is still a new field of application and research. However, it was recognized that new approaches may be necessary in the future to keep pace with the advances in this area [1]. At present, there is a lot of knowledge available to address the hazard and applications of nanomaterials (NMs) in the agri-food sector, however, it is scattered and limited to either particular applications or understandings of NM hazard. The aim of the present review is to integrate the applications and hazards of NMs to plants and critically address the safe use of NMs in the agri-food sector.

Potential applications of nanotechnologies in the agri-food sector include nanopesticides and nanofertilizers, nanozeolites and hydrogels to improve soil quality, NMs (SiO_2_, TiO_2_, and carbon nanotubes (CNTs)) to stimulate plant growth, and smart monitoring using nanosensors in connection with wireless communication devices. In addition, engineered NMs (ENMs) could be used for pesticide degradation, plant germination and growth, crop disease control, water purification, and pesticide residue detection [2]. While many agri-food nanotechnologies appear to be highly promising, they are not yet widely manufactured and implemented [3].

On the other hand, the unintentional emission of NMs due to increasing incorporation into consumer products has raised questions over the short- and long-term effects they may have on plants, i.e., food crop productivity, trophic transfer, and, ultimately, environmental and human health [4].

In this paper, we review recent scientific data on the application of ENMs in agriculture and data on the adverse effects of ENMs on plants in cases of unintentional exposures for some selected ENMs (C-, Ag-, Ce-, Ti-, and Zn-based ENMs). A literature search was made in the ACS, RSC, and Springer publication databases as well as in Google Scholar, using the search terms ‘plant’ AND ‘nanoparticle’ OR ‘nanomaterial’ for the years 2017 and 2018. Articles using non-agricultural and/or non-terrestrial plant species were excluded, along with papers whose topics did not align with those covered in this review. In the first part, we discuss the fate of NMs when interacting with plants (adsorption, internalization, translocation, and bioaccumulation), which is a major contributor to ENM effects, both beneficial and adverse. In the second part, we review adverse versus beneficial effects of some selected ENMs on plants. We address the question of whether the application of ENMs in the agri-food sector is justified from a nanosafety perspective and whether plants face a high hazard potential from ENMs during normal crop cultivation. Our primary aim is to support decision making for the application of ENMs to agricultural plants, including the Safety of Novel Foods and Feeds, which may include ENMs.

## 2. NM Interactions with Plants

A wide range of factors, including plant species, growth medium, exposure route and duration of exposure, abiotic and biotic stressors, and NM physicochemical properties affect plant–NM interactions [5,6]. These factors, along with NM adsorption, internalization, translocation, and bioaccumulation may be major contributors to their effects, both beneficial and adverse (i.e., agricultural crop yields, nutritional quality, NM transfer to human consumers, or plant nanotoxicity). Although plants have always been in contact with natural NMs (e.g., from forest fires and volcanic eruptions), there is significant interest in understanding their interactions with those originating from nanotechnologies [7]. In agricultural settings, plants are likely to be exposed to NMs through the application of biosolids and agricultural nanotechnologies (e.g., fertilizers, pesticides, and growth regulators) and through atmospheric deposition (especially in urban and industrial areas) [8,9]. Summaries of recent articles from 2017 and 2018, which address unintentional ENM exposures and their impacts on agricultural plants, are shown in Table 1.

Recently, Drobne, et al. [10] published a review on the application of different microscopy and spectroscopy techniques for detecting and visualizing NMs in biological samples, which shows developments in the field of studying ENM loads and distributions in biota. The NM load can be transferred to higher levels of the food chain and also consumed by humans when adsorbed to or internalized by edible plants. Retention by leaves may occur by entrapment on the outermost cuticular wax layer and internalization through openings, such as stomata, that regulate gas and water balance. Translocation to roots may occur via phloem transport together with the products of photosynthesis [11]. In roots, NMs can also be internalized with water and nutrients in soil or hydroponic media, with uptake being highly modulated by external factors including the growth medium [12], pH [13,14], cation exchange capacity [15], root exudates [16,17], and mycorrhizal fungi [18,19]. In the event of uptake, translocation to leaves may be restricted by the Casparian strip, necessitating symplastic transport through cellular plasmodesmata to reach the xylem and phloem [4]. For both root and foliar exposures, NM uptake is highly dependent on the plant species and transpiration rate [9,11,20], and NM size [18,21], chemical composition [22,23], surface functionalization [24,25,26], age [27], and stability [28].

## 3. Unintentional NM Exposure and Impacts on Agricultural Crop Yield and Nutritional Value

A number of studies have shown that NMs may have adverse or beneficial effects on agricultural plant yield and nutritional value, which raise important implications for food quality. Summaries of both types of effects from recent literature (2017 and 2018) are shown in Table 1, while further elaboration of these effects is provided in Sections S1–S6 in the Supplementary Materials.

## 4. Co-Exposure to ENMs and Pollutants and Effects on Bioaccumulation and Phytotoxicity

Plant co-exposure to ENMs and environmental contaminants has been recently reviewed [47] and is a growing topic of interest in plant-ENM studies. Engineered NM co-exposures with organic and metal pollutants and other types of ENMs may affect the uptake and translocation of each material by plants, which ultimately affects NM interactions with plants, both adverse and beneficial. Summaries of some additional papers from 2017 and 2018 to those presented in a recent review by Deng et al. [47] are listed in Table 2.

Changes in plant susceptibility to pollutants when co-exposed with ENMs are often attributed to the high adsorption affinities of NMs which can enhance pollutant uptake and subsequent adverse effects. This was reported for rice co-exposure to graphene oxide (GO) and polycyclic aromatic hydrocarbons (PAHs) in hydroponic medium (26% and 92% higher PAH uptake at 0.01 and 0.1 mg GO/L, respectively) [48]. By the same mechanism, pollutant uptake and toxicity may also be decreased if the pollutant concentration and/or bioavailability are reduced due to its immobilization by surface adsorption to ENM surfaces. Deng et al. [25] reported reduced carbamazepine uptake by collard greens in soil and hydroponic medium with co-exposure to pristine and surface carboxylated CNTs, while Liu et al. [49] reported that CuO NMs (50 mg/kg soil) reduced As rice grain content by 35% relative to cultivation with As alone (10 mg/kg soil). In addition, the almost complete elimination of tetracycline uptake in rice was documented in the case of co-exposure with TiO_2_ NMs in hydroponic medium [50]. In soil-cultivated barley plants, SiO_2_ NMs alleviated the negative impacts of NiO NMs on plant biomass and antioxidant activity levels, and completely reversed its effects on photosynthetic parameters [51]. Very recently, Rossi et al. [17] reported that increased soybean root exudate excretion in response to polyvinylpyrrolidone (PVP)-CeO_2_ NMs and Cd^2+^ co-exposure led to binding between biomolecules in the root exudate and Cd^2+^, thus reducing shoot Cd content by 78% in hydroponic medium. In addition, shortened root apoplastic barrier structures in rapeseed following co-exposure to PVP-CeO_2_ NMs and NaCl in sand medium were attributed as the cause of altered Na concentrations in roots (−35%) and leaves (+30%) relative to the individual exposure treatments [52]. When assessing the effects of NMs on plants, it is necessary to take into consideration that co-exposure with other chemicals may significantly modify their effects.

## 5. NM Biotransformation in Plants

Nanomaterial biotransformations are a result of NM-biota interactions and alter the behavior and fate of ENMs in the environment. Nanomaterial biotransformations include dissolution, redox reactions, and chemical reactions with surrounding molecules which occur in contact with biological media and biological surfaces [58]. Some ENMs are generally recognized as stable under environmental and biological conditions, while others are prone to transformations. Table 3 provides summaries of ENM biotransformations recorded in recent literature from 2017 and 2018.

From among the most recent literature on NM biotransformation, two points can be made. The first is that NM uptake and biotransformation are reported to follow dissolution outside the plant tissue. In other words, biotransformation of undissolved NMs does not appear to occur. This was reported for bean root exposure to ZnO NMs in hydroponic medium [59], rice root exposure to CuO NMs in soil [36], and lettuce root exposure to weathered CuO NMs, in which a 214% greater root Cu uptake was reported relative to plants exposed to unweathered CuO NMs [54].

The second point is that NM uptake and biotransformation occur more frequently in hydroponic medium, which is more conducive to NM aggregation and dissolution around roots than soil, indicating that NMs are more likely to exert effects on plants in hydroponic medium. No CeO_2_ NM biotransformation was detected following wheat [34] and tomato and fescue [60] root exposure to CeO_2_ NMs in soil, whereas studies conducted in hydroponic medium showed biotransformation of Ce(IV) to Ce(III) (15–20%) in cucumber [28] and wheat roots [61]. A recent study involving wheat root exposure to Ag NMs or Ag_2_S NMs in hydroponic medium reported complete biotransformation of both NM types, despite the fact that Ag_2_S NMs are reported to be highly stable. Ag_2_S NMs often constitute the final end product for Ag NMs, which may precipitate from Ag^+^ ions before sulfidation into Ag_2_S NMs. However, the lack of metallic Ag and the presence of Ag-thiol complexes (13%) in the secondary root tissue of Ag_2_S NM-exposed plants supported the conclusion that the Ag_2_S NMs dissolved prior to uptake, most likely due to the presence of root exudates [23]. Of relevance to toxicity, a bean seed germination study conducted with ZnO NMs in water reported that it was the amount of biotransformed Zn, rather than the total amount of Zn incorporated into the seedlings, that correlated with the severity of adverse effects (i.e., reduced weight gain) [62]. These findings suggest that under typical outdoor agricultural cultivation in soil, plants are more susceptible to NMs that dissolve easily (e.g., Ag, Cu, and Zn), with the main risk originating from the dissolved ions rather than from the NMs themselves.

## 6. Applications of ENMs in the Agri-Food Sector

There has been substantial interest in harnessing the intrinsic properties of NMs for agricultural applications, which has been the subject of several recent reviews [65,66,67,68]. It is now clear that there is not only one characteristic of NMs responsible for their biological effects, but rather that their interplay with environmental and biological media over time can result in beneficial or adverse effects for agri-food systems. Engineered NMs are used for targeted treatments as fertilizers, antimicrobial agents, and carrier systems for active ingredients (i.e., pesticides, herbicides, fertilizers, growth hormones, and metal nutrients). As carriers, ENMs (typically C-based) can increase the solubility, stability, and bioavailability of active ingredients, reducing field losses from runoff, degradation, and volatilization, and minimizing the extent of downstream environmental pollution [67,69]. Additional applications include seed coatings or soaks and hydroponic additives. A summary of recently published studies focusing on ENM applications in agriculture from 2017 and 2018 is shown in Table 4.

In the case of seed treatments [21,62,70,71,72,73,74,75,76] and early-stage root treatments in hydroponic media [56], ENM transfer into edible plant segments and to the environment is limited. In view of their reported benefits, their use appears to be justified. Relatively few studies investigated the addition of ENMs to soil, however, tested materials include those which are already used in outdoor settings (i.e., CB and hydroxyapatite) [55,77] and those which are prone to dissolution and are commonly used in conventional agriculture in dissolved form (i.e., Cu and Zn) [27,62,70,72,78]. This suggests that such uses are also justified from a nanosafety perspective. All foliar sprays, regardless of their intended function, directly expose plants and the environment to ENMs through drift. Borgatta et al. [78] found that dipping leaves into a NM-containing suspension provided superior results to spraying because it reduced drift while improving leaf exposure. There are no field studies on soil accumulation of these ENMs, but, with frequent use, in parallel environmental monitoring should be required.

## 7. Hazard Potential of ENMs to Plants: General Perspective

**Figure 1:**
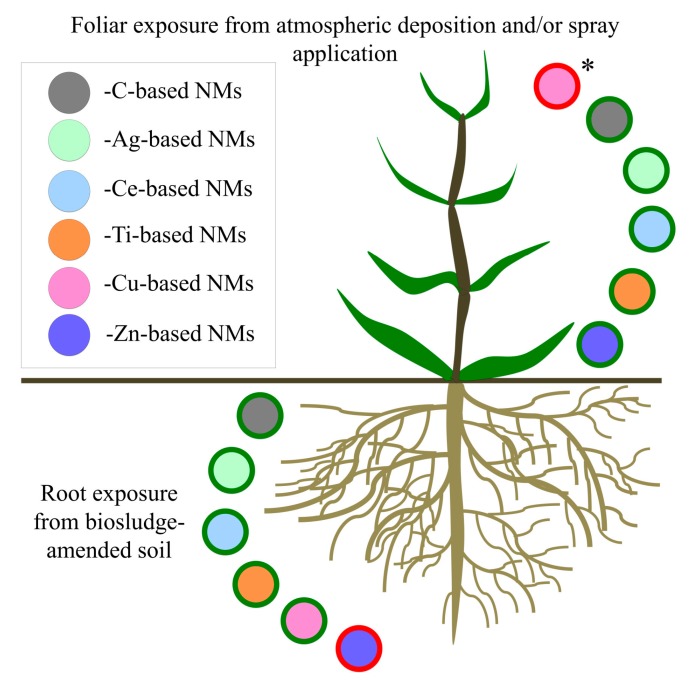
Graphical representations of commonly investigated ENM types and their hazard potential to agricultural plants (green and red outlines represent low and high hazard potential, respectively). * Potential toxicity from foliar application of Cu-containing foliar sprays and atmospheric deposition in urban areas.

A summary of NOAEL (no observed adverse effect level) and LOAEL (lowest observed adverse effect level) values for plants exposed to the most frequently studied ENMs in the recent literature from 2017 and 2018 (C-, Ag-, Ce-, Cu-, Ti-, and Zn-based ENMs), together with their corresponding concentrations in relevant environmental matrices, are supplied in Table 5, while a graphical representation of the general hazard potential from these ENMs is available in Figure 1.

Risk analyses based on comparison of ENM LOAEL values with their concentrations in waste water treatment plant (WWTP) biosludge and biosludge-amended soil shows that, with a few exceptions which include dissolving ENMs, there is a low risk of plant phytotoxicity (Table 5). Mean concentrations of Ag- and Ce-based ENMs in biosludge-amended soil (assuming the unlikely scenario of 100% ENM persistence) were approximately 400–20,000 times lower and 200–11,000 times lower, respectively, than their lowest LOAEL values for root exposure in soil [84]. As TiO_2_ ENMs had no adverse plant effects up to a concentration of 750 mg/kg in soil, it is unlikely that a biosludge concentration of 170 mg/kg poses a risk to plants [85]. Among C-based ENMs, CNTs and CB (which exhibited an inverse dose-response relationship of greater toxicity at lower concentrations) [86] pose a low hazard potential, as they were measured at concentrations approximately 1.5 times and 5000–50,000 times above the LOAEL value of CNTs and CB in biosludge, respectively [85,87]. Even where adverse effects increased with increasing concentration in soil [88], the LOAEL concentration for CNTs is roughly 300 times below the concentration of CNTs measured in biosludge [85] and therefore would not have a high hazard potential. With Cu-based ENMs, their concentration measured in biosolids was ~200–5000 times lower than the lowest LOAEL value for root exposure in soil, indicating a nearly non-existent risk of phytotoxicity [87]. However, plants were adversely affected by short-term foliar exposures to CuO NMs [9] and a Cu(OH)_2_ pesticide spray, therefore this route of exposure could present a hazard to plants [38,89]. Only Zn-based ENMs were measured in biosludge at higher concentrations than the lowest level that induced toxicity from root exposure in soil (8 times higher) [84,85,87]. However, soil characteristics were shown to play a more dominant role than that of ZnO NM exposure in plant responses, and the reported adverse effects were changes to antioxidant enzyme activity levels rather than to physiological growth parameters [14].

There remains a lack of understanding about plant responses to long-term, low-dose exposures to ENMs, including exposures that occur over successive plant generations. Therefore, it is not possible to accurately model the future severity and types of effects that might occur as a result of current agricultural plant exposures to ENMs. Rather, we propose that environmental and biological monitoring (biomonitoring) would present an acceptable solution for recording plant exposures to ENMs before other regulatory requirements are elaborated and put into practice. Wastewater treatment plant biosludge and biosludge-amended soils are ideal matrices for environmental monitoring because they form the main point of agricultural plant exposure to ENMs originating from consumer and industrial sources [8,90]. Biomonitoring goes further by providing information on the bioavailability of a given substance through the measurement of specific biomarkers in target species [90]. A number of ENM-specific plant biomarkers have been identified in a recent meta-analysis of literature on omics-level plant responses to ENMs by Ruotolo et al. [91], indicating promising advances in this area.

## 8. Conclusions and Future Perspectives

Through a review of the recent literature, we have shown that agricultural plants do not currently face a high hazard potential from ENMs during crop cultivation. Many of the most frequently investigated ENMS (C-, Ce-, Ti-, and Ag-based) are present in environmental media at concentrations that are unlikely to pose a significant threat to agricultural plant safety, while Cu- and Zn-based ENMs may have the potential to exert adverse effects, depending on the mode of exposure and soil characteristics, respectively. A number of key points can be made:

- NMs do not pose risks to plant safety and agronomic characteristics, such as yield and nutritional quality, except at extremely high, environmentally unrealistic concentrations;
- NM dissolution appears to be a significant driver of toxicity due to the increased bioavailability of ions;
- NM co-exposures may enhance or diminish the risks posed by other toxic pollutants;
- NMs at low concentrations and/or applied during the early stages of plant growth (e.g., as seed coatings) provide beneficial effects with limited introduction into the environment or edible plant segments, justifying such uses from a nanosafety perspective.

In order to make progress in anticipating and responding to plant-ENM exposures and promote the responsible use of agricultural nanotechnologies, numerous data gaps must be addressed in future research. Multi-generational plant exposure studies that simulate realistic field exposure conditions and ENM types and doses are greatly needed, especially for evaluating ENM-based agricultural products which are already on the market. Likewise, ENM co-exposure and biotransformation studies remain needed to better understand the persistence and uptake of ENMs and other substances (e.g., nutrients and contaminants) which may be present in soil. While agri-food nanotechnologies have a high potential to reduce environmental pollution and ecosystem and human health risks associated with conventional agricultural practices while increasing food production and quality, the already-listed limitations and knowledge gaps make it difficult to compare the use of nanotechnologies with conventional practices in terms of these factors. Despite these informational gaps in the understanding of plant responses to ENMs, the implementation of effective monitoring for ENMs in the environment and plant responses to them (biomonitoring) could help to assure their beneficial use in the agri-food sector.

## Figures and Tables

**Table 1:** Summaries of engineered nanomaterial (ENM) effects on agricultural plant yield and/or nutritional contents documented in recent papers from 2017 and 2018.

NM	Size	Plant Species	Exposure/Medium	Duration	Results	Reference
**Carbon-Based NMs**						
CNO	20–40 nm	Gram	Sprouted seed; 0, 10, 20, and 30 µg/mL water. Transfer to soil after 10 days.	10 days; harvested after ~4 months	Increased protein, electrolytes and micronutrients, size, and weight of mature seeds without CNO uptake	[29]
Chitin	80–200 nm long, 30–50 nm wide	Winter wheat—MSW and LSW cultivars	Seed, root; 0, 0.002, 0.006, and 0.02 g/kg sandy soil	Full life-cycle	Increased grain protein, Fe, and Zn contents Improved photosynthetic parameters for both cultivars (0.006 g/kg for MSW)	[15]
MWCNTs	15–40 nm wide	Barley Maize Soybean	Root; 50 µg/mL deionized water with nutrient solution	20 weeks	Significantly longer shoot growth in maize and barley and decreased root biomass in soybean and maize 10% increased photosynthetic capacity in maize	[30]
**Metal-based NMs**						
Ag	20 nm	Peanut	Seed and root; 50, 500, and 2000 mg/kg sandy soil	98 days	Ag NMs internalized in a dose-dependent manner and significantly reduced plant growth parameters and yield fatty acid composition in edible peanut grains was adversely affected	[31]
Ag	5.6 nm	Wheat	Seed and root; 20, 200, and 2000 mg/kg soil	4 months	Significantly reduced plant growth and biomass (all doses) Increased grain Ag (200 and 2000 mg/kg) and reduced grain Fe (2000 mg/kg), Zn, and Cu (200 and 2,000 mg/kg) Reduced yield and grain protein and amino acid contents (200 and 2000 mg/kg)	[32]
Ag with PEG coating	7–14 nm	Tomato	Root; 10 mg/kg soil	56–62 days	Reduced NPK uptake, chlorophyll content, fruit yield; increased fruit Ag	[33]
CeO_2_	8 ± 1 nm	Wheat	S1 plants grown to maturity (0, 125, and 500 mg/kg soil); seeds grown in factorial combinations (1, 125, and 500 mg/kg) (S2 plants).	90 days	Decreased root Ce, Al, Fe, and Mn concentrations and improved physiological characteristics of S2 plants produced from treated S1 plants Consecutive S1 and S2 exposures adversely affected grain nutrient quality (125 mg/kg) or growth parameters (500 mg/kg)	[34]
CeO_2_	15 ± 5 nm	Sorghum	Foliar: 0 and 2 mg/plant applied 60 days after sowing, at which time one group was subjected to drought conditions for 21 days. Soil medium used.	>21 days (until maturity)	Lower lipid peroxidation and increased photosynthetic rates and seed yield per plant (31%) in unwatered, exposed plants relative to the unwatered control	[35]
CuO	43 ± 9 nm	Rice	Root; 50, 100, 500, and 1,000 mg/kg soil	7, 21, 60, and 88 days	Physiological parameters and grain yield adversely affected (500 and 1000 mg/kg) Grain Cu, Zn, and Fe were greatly elevated in mature plants (500 mg/kg)	[36]
CuO	20–100 nm	Bell pepper	Root; 0, 125, 250, and 500 mg/kg soil	90 days	Root Cu concentrations were elevated compared to the control (250 and 500 mg/kg); reduced nutrient uptake to fruits and leaves	[37]
CuO	40–60 nm	Lettuce Cabbage	Leaf; 0, 10, and 250 mg/plant (applied as dry particles to adaxial surfaces); plants grown in soil medium	5, 10, and 15 days	Lettuce dry weight increased at 10 mg/plant and decreased at 250 mg/plant Phytotoxic effects for both plants (250 mg/plant)	[9]
Cu(OH)_2_	(~50 -> 1,000 nm)	Spinach	Leaf; 0, 1.8, and 18 mg/plant; plants grown in artificial growth medium	7 days	No change to biomass or photosynthetic pigment contents Reduction of beneficial antioxidant compounds and amino acids and increase in the primary products of photosynthesis (18 mg/plant)	[38]
TiO_2_	20–100 nm	Rice	Root; 50 and 200 mg/kg soil under background or elevated CO_2_ (370 and 570 µmol mol^−1^, respectively)	130 days	Treated plants had decreased grain yield and plant biomass compared to control plants (high CO_2_) Rice grains (200 mg/kg) had reduced fat, protein, and total sugar contents (high CO_2_) and increased reducing sugar, Ti, P, Mg, Ca, Mn, and Zn grain contents with increasing NM treatment (high CO_2_)	[39]
TiO_2_	20 nm	Rice	Seed, root: 0, 25, 50, 150, 250, 500, and 750 mg/kg P-deficient soil	Full life-cycle	TiO_2_ NM addition increased P uptake and plant growth (50–750 mg/kg) without translocation to grains	[40]
ZnO	18 nm	Winter wheat	Root; Fresh soil with 6 mg/kg soil and used soil with 5.98 mg/kg (previously used to grow sorghum and aged for 6 months)	Grown to maturity	Leaf chlorophyll levels and shoot height increased in used soil; biomass unaffected Grain yield and Zn content increased in used and fresh soil	[41]
ZnO	18 nm	Sorghum	Root; 6 mg/kg soil Leaf; 100 mL treatment with same amount of Zn as in soil. Low or high soil N, P, and K for root and leaf exposures	Not provided	Increased grain yield and grain Zn, N, K, and P under all experimental variations	[42]
ZnO	<100 nm	Bean Tomato	Root; 3, 20, 100, and 225 mg/kg acidic (pH 5.4) or calcareous (pH 8.3) soil	90 days	Increased photosynthetic pigments and increased protein in calcareous soil and higher leaf Zn in acidic soil	[14]
ZnO	30 nm	Maize	Root; 0 and 500 mg/kg soil with and without organic P (0, 20, and 50 mg/kg) and AMF (*Funneliformis* mosseae; with and without)	9 weeks	ZnO NMs increased root dry weight of inoculated plants (0 and 50 mg P/kg) Inoculated plants exposed to ZnO NMs and P had less Zn in shoots and roots than uninoculated plants Inoculated plants exposed to ZnO NMs had increased shoot Mn and root Mn and Cu	[43]
ZnO (bare and hydrophobically-coated)	93.8 nm (bare) 84.1 nm (coated)	Bean	Root; S1 plants grown in soil with 125, 250, and 500 mg/kg soil. S2 plants grown in soil without NMs	Grown to maturity	No differences in the number, weight, and sugar, starch, and protein contents of S2 seeds compared to the other groups Reduced Ni content of S2 seeds with both NM types	[44]
ZnO (bare and hydrophobically-coated)	10–300 nm	Bean	Seed and root; bare and hydrophobically-coated NMs (62.5, 125, 250, and 500 mg/kg) in natural soil (NS) and organic matter-enriched soil (ES)	>45 days, until maturity	Seed yield and nutrients (Zn, Fe, Mg, Ca, Fe, and Mn) were greater in ES compared to NS No differences compared to bulk and ionic formulations	[26]
ZnO B_2_O_3_ CuO	<100 nm <100 nm <50 nm	Soybean	Leaf; 20 mL with all 3 NMs (1.77 g ZnO/L, 0.80 g CuO/L, and 0.92 g B_2_0_3_/L water) followed by a 14 day drought period	19 weeks	Increased grain count (number/plant), grain dry weight (g/plant), and grain N and K with respect to the control at physiological maturity	[45]
Fe_2_O_3_ CuO TiO_2_	20 nm 40 nm 5 nm	Peanut	Seed and root; 50 and 500 mg/kg soil	145 days	1000-grain weight decreased across all treatments; per plant yield decreased only at 500 mg/kg Except for the 50 mg TiO_2_/kg treatment, total amino acid contents of peanut grains were decreased across all treatments (12.0%–33.6%) Resveratrol content increased (all treatments)	[46]

AMF = arbuscular mycorrhizal fungi; CAT = catalase; CNO = carbon nano-onion; ES = organic matter-enriched soil; GS = glutamine synthetase; GOGAT = glutamate synthase; LSW = large spike wheat; MSW = multi spike wheat; MWCNT = multi-walled carbon nanotube; NOM = natural organic matter; NR = nitrate reductase; NS = natural soil; POD = peroxidase; SOD = superoxide dismutase; TF = translocation factor.

**Table 2:** Summaries of recent ENM co-exposure studies from 2017 and 2018.

NM	Size	Plant Species	Exposure/Medium	Duration	Results	Reference
GO	2.0 ± 0.5 nm wide, 0.5–5 µm long	Rice	Root; 0.01, 0.1, and 1.0 mg/L ½-strength culture solution with or without 10 µg/L PAHs	7 days	GO at low concentrations (0.01 and 0.1 mg/L) increased PAH root uptake (26.4–92.5%) and ROS GO at 1.0 mg/L decreased PAH uptake and ROS compared to the control treatments	[48]
pCNTs cCNTs	<8 nm wide; 10–30 µm in length	Collard greens	Root; 50 mg/L hydroponic medium and 500 mg/kg soil with carbamazepine (100 µg/L and 100 µg/kg, respectively)	28 days (hydroponics); 42 days (soil)	Both NM types reduced plant carbamazepine concentrations and translocation from roots to leaves The carbamazepine TF from roots to leaves was higher for cCNTs than for pCNTs in hydroponics	[25]
MWCNTs	36.5 ± 12.7 nm width; 350 nm length	Rice Maize Soybean	Root; 2.25 mg/L with SPAOMs (0 and 0.325 mM) in ½-strength Hoagland solution.	1 day	MWCNTs reduced antioxidant enzyme activities that were increased by exposure to SPAOMs alone Co-exposure increased proteins levels that were reduced by individual SPAOM and MWCNT treatments	[20]
PVP-CeO_2_	41.7 ± 5.2 nm	Soybean	Root; 0 and 500 mg/kg sand with 25% Hoagland solution with Cd (0, 0.25, and 1 mg/kg sand)	30 days	Total biomass was decreased with Cd (1 mg/kg) and root biomass remained decreased with CeO_2_ NMs Co-exposure significantly increased Ce uptake by roots and older leaves (NMs + 1.0 Cd) relative to plants cultivated only with CeO_2_ NMs but did not affect Cd internalization	[53]
PVP-CeO_2_	41.7 ± 5.2 nm	Soybean	Roots; 100 mg/L tap water with and without 1.0 mg Cd^2+^/L tap water	8 days	No significant changes to dry weight Co-exposure reduced root Ce (45%) but increased shoot Ce (44%) compared with NM exposure alone Cd uptake in shoots was reduced (78%) with co-exposure relative to Cd exposure alone	[17]
PVP- CeO_2_	52.6 nm (average)	Rapeseed	Root; 0 and 500 mg/kg dry sand and NaCl (0 and 50 mM)	3 weeks	Reduced biomass from CeO_2_ NM+NaCl and NaCl alone Co-exposure altered Ce and Na concentrations relative to treatment with NMs and NaCl alone Co-exposure resulted in shortened apoplastic barriers near the root apex relative to NaCl alone	[52]
CuO	23–37 nm	Rice	Seed; 0, 0.1, 1.0, 10, 50, and 100 mg/L in 20% Hoagland solution for 18 days Root; soil medium with and without as (0 and 10 mg/kg)	131 days	CuO NMs and as alone increased total grain dry weight 17–25% and 13%, respectively CuO NMs + As increased total grain dry weight (0.1 and 1 mg CuO/L, respectively) relative to CuO NMs alone CuO NMs+As treatment reduced grain as by 35% compared to As treatment alone (50 mg CuO/L)	[49]
CuO	40 nm	Lettuce	Root; Pristine and weathered (mixed with soil 70 days prior to use) NMs at 0 and 400 mg /kg soil co-contaminated with chlordane (concentration not provided)	70 days	NM weathering led to increased root Cu uptake (214%) compared to treatment with pristine NMs Significantly decreased biomass for both NM treatments compared to the control Weathered NM treatment significantly increased chlordane uptake relative to bulk and ionic Cu forms	[54]
BC Ni/Fe BC-supported Ni/Fe	28.4 nm (Ni/Fe NMs)	Chinese cabbage	Seed and root; 0 and 30 mg/kg soil contaminated with BDE209	20 days	Harmful effects of BDE209 were most alleviated by treatment with the BC treatment, followed by BC-Ni/Fe and Ni/Fe.	[55]
nHAP	20 ± 5 nm	Rice	Root; 100 mg/L Hoagland solution (5 days) followed by movement into solution with Pb(NO_3_)_2_ (0, 15, and 25 µM; 14 days)	19 days	nHAP pre-treatment reduced the toxic effects of Pb on plant growth and Pb translocation from roots to shoots	[56]
SiO_2_	25 nm	Barley	Seed and root; 3 mg/kg artificial soil with or without 120 mg NiO NMs/kg	14 days	Exposure to SiO_2_ significantly reversed the negative impacts of NiO NMs on leaf and root fresh weights and completely reversed negative impacts on photosynthetic parameters	[51]
TiO_2_ (≥99% anatase)	10–25 nm	Rice	Root; 500, 1,000, and 2,000 mg/L with tetracycline (TC; 0, 5, 10, and 20 mg/L) in ½-strength Hoagland solution	10 days	TiO_2_ NMs and TC alone reduced root and shoot growth with increasing concentration; co-exposure improved growth Co-exposure reduced TC uptake and antioxidant enzyme activities and reversed nutrient deficiencies (P, S, and Zn) from TC alone	[50]
ZnO CuO Cr_2_O_3_TiO_2_ Fe_2_O_3_	100 ± 25 nm 50 ± 10 nm 100 ± 30 nm 25 ± 6 nm 50 ± 15 nm	Cress Flax Wheat Cucumber	Seed; dispersions contain each NM singly or in combinations (ZnO or CuO NMs with Cr_2_O_3_, TiO_2_, or Fe_2_O_3_ NMs) at 10, 100, and 1,000 mg/L redistilled water	3 days	Plant root length slightly less affected by paired NM treatments than by individual treatments for all plants except in the cases of treatment with CuO + ZnO NMs and CuO+Fe_2_O_3_ NMs_,_ where root toxicity was significantly decreased	[57]
ZnO CeO_2_La_2_O_3_CuO CdS QDs	<100 nm <25 nm 10-100 nm 40 nm <5 nm	Zucchini	Root; vermiculate with dispersion containing each NM individually and in binary combinations (500 mg NMs/L; 100 mg QDs/L)	21 days	Combined treatments generally reduced biomass relative to individual treatments (except for CdS QD treatment) Metal concentrations from combined treatments were generally the same as in individual treatments	[22]

BDE209 = decabromodiphenyl ether; QD = quantum dot; TOC = total organic carbon.

**Table 3:** Summaries of ENM biotransformation studies in plants from 2017 and 2018.

NM	Size	Plant Species	Exposure/Medium	Duration	NM Biotransformations	Reference
Metal-Based NMs						
Ag Ag_2_S	52 ± 1 nm 42 ± 5 nm	Wheat	Root; 30 µM Ag or Ag_2_S NMs in ¼-strength Hoagland solution	3 weeks	Ag was completely dissolved and complexed by thiols (86%) and ionic species (14%) in root tissue Ag_2_S was dissolved and reduced to elemental Ag and complexed by thiols (13–26%) in secondary root tissue	[23]
Ag_2_S	59 nm	Wheat Cucumber	Root; 20 mg/L nutrient solution	7 days	Little biotransformation (1–9% associated with glutathione)	[63]
CeO_2_	25.2 ± 2.3 nm	Cucumber	Root; split root hydroponics system (ultrapure water)—one half placed in 200 and 2000 mg CeO_2_/L; other half in ultrapure water	3 days	Biotransformation occurred only at the root surface (~15% of Ce(IV) reduced to Ce(III) in the treated root half) 18% and 8.1% of Ce was present as Ce(III) in the shoots (200 and 2000 mg/L, respectively) Only Ce(IV) was identified in the unexposed root half	[28]
CeO_2_	8 ± 1 nm	Wheat	S1 plants were grown to maturity (0, 125, and 500 mg/kg soil) and the seeds were cultivated in factorial combinations (1, 125, and 500 mg/kg) to maturity (S2 plants).	90 days	Ce was not internalized and did not change speciation (Ce^4+^to Ce^3+^) in soil or on the root surface	[34]
CeO_2_	Length: 67 ± 8 nm Diameter: 8 ± 1 nm	Barley	Root; 250 mg/kg soil	60 days	Ce detected on root surfaces mostly as CeO_2_ (84–90%) with smaller amounts of Ce(III)(10–16%) with almost no uptake Hotspots of Ce(III) were detected on areas of the root surface where CeO_2_ was also found to be internalized	[64]
CeO_2_ (bare and citrate-coated)	3 ± 1 (bare) 3.9 ± 1.8 nm (citrate coated)	Fescue Tomato	Root; 1, 15, or 50 mg bare or citrate coated CeO/kg of either sandy soil with low NOM or clay-rich soil with high NOM	Not provided	Chemical stability was confirmed, regardless of surface coating and soil type	[60]
CeO_2_ (3 surface coatings)	4 nm	Wheat	Root; 20 mg/L ¼-strength Hoagland solution containing NMs functionalized with neutral, positive, or negative charge	34 h	Ce(IV) was reduced to Ce(III) (15–20%) in roots and leaves, regardless of surface charge Non-vascular areas of leaf tissue with lower Ce concentrations showed higher concentrations of Ce(III)	[61]
CuO	25, 40, and <80 nm	Bean	Seed: 1, 10, 100, and 1000 mg Cu/L of aqueous medium for each size separately	5 days	The speciation of 40 nm NMs inside the embryos was reduced compared to 40 nm NMs in the seed coat or outside the seed (34 ± 1% Cu_2_O and 66 ± 1% CuO) No change in speciation of 25 and 80 nm NMs	[21]
CuO	43 ± 9 nm	Rice	Root; 50, 100, 500, and 1000 mg/kg soil	7, 21, 60, and 88 days	In rice, most Cu was in the form of Cu-citrate and about a third of Cu(II) was biotransformed to Cu(I) (mainly associated with cysteine) In soil, all CuO was transformed to Cu_2_S and Cu adsorbed to goethite (about a third of total Cu) at maturation	[36]
CuO	40 nm	Lettuce	Root; Pristine and weathered (mixed with soil 70 days prior to use) NMs at 0 and 400 mg /kg soil	70 days	Weathered NMs were completely reduced to Cu_2_O and Cu_2_S in root tissue In weathered treatments, the percentage of Cu_2_S was higher in secondary and main root tissues than in epidermal tissue or aggregates on the root surface Unweathered NMs were present in oxidized and reduced form in the epidermis and secondary root	[54]
CuO	40–60 nm	Lettuce Cabbage	Leaf; 0, 10, and 250 mg/plant (applied as dry particles to adaxial surfaces); plants grown in soil medium	5, 10, or 15 days	Various biotransformations likely occurred, including Cu(0) to Cu-organic complexes Cu(II) reduction to Cu(I), likely from Mn-mediated electron transfer, reflecting oxidative stress from CuO NM exposure	[9]
ZnO	20, 40, and 60 nm	Bean	Seed; 1, 10, 100, 1,000, and 5,000 mg/L deionized water for each NM size	20 min; harvested after 5 days	The inner and outer seed coats of control seeds contained Zn-histidine whereas the seed coats of treated seeds contained mixtures of ZnO and Zn-malate In addition to ZnO, Zn-histidine, Zn-malate, and Zn-citrate were identified within treated and control seeds	[62]
ZnO	20, 40, 60, and 300 nm	Bean	Root; 100 and 1000 mg/L aqueous medium with 20, 40, and 60 nm NMs (no surfactant) and 20, 40, and 300 nm (with surfactant)	48 h	In stems, Zn was mainly found as Zn-malate In roots, Zn was found as Zn-malate, Zn-citrate and Zn-histidine	[59]

**Table 4:** ENMs for agricultural applications described in recent literature from 2017 and 2018.

NM	Plant Species	Exposure/Medium	Duration	Aim of Application	Nano-/Commercial Advantage?	Reference
**Carbon-Based NMs**						
ALG/CS CS/TPP	Bean	Seed; 1 h (ALG/CS in 11 mM CaCl_2_ and CS/TPP in 0.1% TPP) with or without encapsulation of gibberellic acid (GA_3_; 0.05%, 0.037%, 0.025%, and 0.012% in distilled water).	1 h; harvested 7 days later	ALG/CS carrier best promoted GA_3_ uptake, leading to increased leaf area and chlorophyll and carotenoid content Both NM types significantly increased plant growth, acting as carrier systems for enhanced stability, solubility, and bioavailability of GA_3_	Yes	[75]
CNO	Gram	Sprouted seed; 0, 10, 20, and 30 µg/mL water. Transfer to soil after 10 days.	10 days; harvested at maturity (~4 months)	Improved seed yield and nutrient contents compared to the control High CEC improves nutrient bioavailability	NA	[29]
Zein NM-GRL, NM-R-CTL	Bean Tomato	Seed; Zein NMs, NM-GRL, and NM-R-CTL (0.05, 0.5, and 5 mg/mL agar medium)	5 days	Encapsulation of geraniol and R-citronellal by zein NMs increased their stability and release, and mitigated plant phytotoxicity (0.05 and 0.5 mg/mL) caused by geraniol and R-citronellal alone	Yes	[74]
**Metal-based NMs**						
BC Ni/Fe BC-supported Ni/Fe	Chinese cabbage	Seed and root; 0 and 30 mg/kg soil contaminated with decabromodiphenyl ether (BDE209)	20 days	High pollutant sorption and complexation by BC reduced the harmful effects of BDE209 due to BC’s high surface area, porosity, and presence of surface functional groups	Yes	[55]
Cu-CNFs	Gram	Seed and root; 10–500 µg/mL aqueous medium	20 days	Enhanced plant growth, water uptake capacity, chlorophyll, protein, and Cu content Improved osmotic conditions for increased water capacity by seeds	Yes	[79]
Cu-chitosan	Maize	Seed; 0.01, 0.04, 0.08, 0.12, and 0.16%, *w*/*v* (4 h) Foliar; corresponding concentration until 35 days old	95 days	Enhanced plant growth and chlorophyll NMs are trapped in chitosan pores, leading to controlled Cu release; chitosan reduces microbial activity through interaction with cell surfaces and DNA/RNA	Yes	[80]
CuO	Bean	Seed: 1, 10, 100, and 1000 mg Cu/L of aqueous medium for each size separately (25, 40, and <80 nm)	5 days	Mass gain was associated with larger particle sizes (<80 nm and 40 nm compared to 25 nm) and lower concentrations (1–100 mg/L for <80 and 40 nm NMs; 1–10 mg/L for 25 nm NMs) High surface area of NMs results in greater control of Cu ion availability	Yes	[21]
CuO	Wheat	Root: ~500 mg/kg soil CuO NMs were either ‘fresh’ or ‘aged’ (added to soil 28 days before exposure)	14 days	CuSO_4_ was more toxic to plants than CuO NMs, despite lower doses CuO NMs were more concentrated around roots than CuSO_4_, providing more targeted treatment	Yes	[27]
CuO NPs Cu_3_(PO_4_)_2_•3H_2_O nanosheets	Watermelon	Greenhouse experiments: Foliar: (1) dipped (0.6–0.8 mL; 10, 50, 100, 250, 500, and 1000 mg/L water) (2) sprayed 1 time (50, 500 mg/L); 3) sprayed 2 times (20, 200 mg/L) Root: 500 and 1000 mg/L Plants cultivated in soilless mix with *Fusarium oxysporum.* Field experiments: Foliar: 400 mg/L; with and without *F. oxysporum* in soil	5 weeks	Greenhouse: foliar-applied nanosheets were more effective at suppressing *F. oxysporum* than NPs when dipped into suspension Foliar-sprayed nanosheets and NPs decreased disease progression compared to the control Similar rates of disease suppression were measured for nanosheets and NPs in field experiments Higher efficacy of nanosheets relative to NPs when leaves were dipped was attributed to the sheet structure and higher initial ion release	Yes	[78]
CuS (3 surface coatings)	Rice	Seed; fungi-infested seeds placed in dispersions containing CuS NMs with 3 coatings: PVP, GABA 4-aminobutyric acid), and citrate (tri-sodium citrate) at 3, 5, 7, 10, and 15 µg/mL	1-2 h; harvested 10 days later	Citrate-coated CuS NMs (7 µg/mL) reduced seed rot and seedling blight and showed enhanced effectiveness CuS NMs may adsorb onto microorganism cell walls, inhibiting their growth	Yes	[76]
Cu/Zn	Winter wheat Stolichna and Acveduc ecotypes	Seed; 1:100 ratio of solution to water, followed by planting in sand medium with water. 8 days after emergence: plants subjected to drought conditions or normally watered for 3 days.	4 h; harvested 11 days after seedling emergence	Seed treatment with NM solution alleviated negative effects of drought in terms of chlorophyll and carotenoid content, TBARs content, antioxidant enzyme activity, leaf area, and relative water content	NA	[81]
GO-Ag	Rice	Seed; Ag NMs and GO-Ag at 1.25, 2.5, 5, and 10 µg/mL ultrapure water. Innoculation with bacterial leaf blight (*Xanthomonas oryzae* pv. *Oryzae* [*Xoo*]).	6 days	*Xoo* was inhibited by GO-Ag NMs at lower concentrations (above 2.5 µg/mL) relative to Ag NMs (10 µg/mL) with less phytotoxicity to germinating seedlings GO decreases Ag oxidation and dissolution; GO sheet morphology provides targeted activity by wrapping around bacteria	Yes	[73]
GO Fe_3_O_4_ GO- Fe_3_O_4_ NMs	Grapevine	Leaf; Plants infected with *Plasmopara viticola* were sprayed with GO, Fe_3_O_4_, and GO-Fe_3_O_4_ at 0 and 250 µg/mL	7 days	GO-Fe_3_O_4_ exerted the highest protective effect on infected leaves GO carrier prevents NM agglomeration. Sporangium germination is inhibited by water channel blockage	Yes	[82]
HA(+) HA(−) HA(0)	Sunflower	Root; 150 mg/kg of each type of HA in two types of P-deficient soil (Ultisol and Vertisol).	35 days	Ultisol soil: all HA types increased plant height and biomass relative to the control in the order (highest to lowest): HA(−) > HA(0) > HA(+) Vertisol soil: increased height and biomass was only measured for plants exposed to a commercial P fertilizer	Yes (Ultisol soil) No (Vertisol soil)	[77]
CNAD-MSNPs	Common pea	Seed; coated with alginate or alginate-CNAD-MSNPs (2 mg/mL MS agar medium); inoculated with *Pseudomonas syringae* pv. *Pisi* (pea blight; OD_600_ = 0.025)	20 days; harvested after 4 weeks in soil	Treatment reduced the rate of infection (28.21% vs. 50% among controls) and improved physiological parameters (larger pods, greater mass, and longer roots) MSNPs protect the CNAD from degradation and run-off, providing the same protection as free CNAD at a dose ~90,000 times lower	Yes	[71]
MSNs	Cucumber	Leaf; 0.5 mL of suspension (200 and 1000 mg/L deionized water) applied to the middle leaf after emergence of the 5th leaf	14 days	MSNs bind spirotetramat (a pesticide), protecting it from degradation and enhancing leaf retention	Yes	[83]
ZnO	Bean	Seed; 1, 10, 100, 1000, and 5000 mg/L deionized water for three different sizes (20, 40, and 60 nm)	20 min; harvested after 5 days	The germination rate was unaffected by the treatments Weight gain was more affected by concentration (decreased at 1000 and 5000 mg/L) than by NM size (from highest to lowest weight gain: 40 nm > 60 nm > 20 nm) Ions are released from NMs at a more optimal rate relative to salts and bulk formulations	Yes	[62]
ZnO (bare, with a Zn_3_(PO_4_)_2_ shell, DEX-coated, and DEX-(SO_4_) coated)	Wheat	Seed; 100, 500, and 1000 mg Zn/L deionized water	24 h; harvested when >65% of control seeds had radicle root at least 20 mm long	Plants exposed to Zn with a Zn_3_(PO_4_)_2_ shell had the highest root mass (67% greater than the control at 500 mg Zn/L) DEX-ZnO NPs increased shoot biomass	Yes	[70]
ZnO MWCNTs ZnO/MWCNTs nanocomposite	Onion	Seed; MWCNTs and ZnO/MWCNTs at 0, 2, 5, 10, 15, 20, and 40 µg/mL and ZnO at 20 µg/mL; seeds germinated under varying watering schedules (every 2nd or 4th days or after the 6th, or 8th day)	20 h; harvested after 12 days	ZnO/MWCNTs increased the germination percentage for seeds watered after the 6^th^ or 8^th^ day but decreased the germination percentage with more frequent watering relative to the other treatments Maximum root and shoot lengths were measured for seeds exposed to MWCNTs and ZnO/MWCNTs (15 µg/mL) MWCNTs provide a scaffold for Zn for controlled Zn ion release and enhance seed water uptake	Yes, for the nano-composite under arid conditions	[72]

ALG/CS = alginate/chitosan; BDE209 = decabormodiphenyl ether; CEC = cation exchange capacity; CS/TPP = chitosan/tripolyphosphate; CNAD-MSNP = cinnamaldehyde-loaded mesoporous silica nanoparticles; CNF = carbon nanofiber; DEX = dextran; HA(+/−/0) = hydroxyapatite with positive, negative, or neutral charge; MS = Murashige and Skoog; MSN = mesoporous silica nanoparticles; NA = not applicable; nHAP = nano-hydroxyapatite; NM-GRL = zein NMs loaded with geraniol; NM-R-CTL = zein NMs loaded with R-citronellal; NP = nanoparticle; OD_600_ = optical density measured at a wavelength of 600 nm.

**Table 5:** Plant NOAEL and LOAEL values for exposure to C-, Ag-, Ce-, Cu-, Ti-, and Zn-based ENMs with the recorded adverse physiological and/or biochemical effect(s).

NM Material	Plant	Exposure Period/Route/Medium	NOAEL	LOAEL	Measured Adverse Effect(s)	Reference
**Carbon-Based ENMs**						
C_60_	Rice	30 day exposure in soil	NA	50 mg/kg	• Reduced root and shoot lengths; increased SOD activity	[88]
CB	Soybean	Up to 41 days root exposure in soil	1000 mg/kg	0.1 and 100 mg/kg	• Reduced plant growth, root nodulation, and N2 fixation potential.	[86]
Chitin	Wheat (MSW and LSW cultivars)	Full life-cycle root exposure in sandy soil	0.02 g/kg	NA		[15]
CNOs	Gram	10 day sprouted seed exposure in water before transplantation to soil	30 µg/mL	NA		[29]
CNTs (carboxylated)	Collard greens	42 days root exposure in soil	500 mg/kg	NA		[25]
GNPs	Soybean	Up to 41 days root exposure in soil	100 mg/kg	mg/kg	• Reduced plant growth.	[86]
GO	Oats	15 days in vermiculite	40 mg/L	200 mg/L	• Reduced chlorophyll contents and increased MDA activity	[92]
rGO	Rice	30 day exposure in soil	NA	50 mg/kg	• Reduced root and shoot lengths and shoot dry weight; increased SOD and POD activities	[88]
MWCNTs	Rice	30 day exposure in soil	NA	50 mg/kg	• Reduced root and shoot lengths; reduced SOD and POD activities	[88]
MWCNTs	Soybean	Up to 41 days root exposure in soil	NA	0.1 mg/kg	• Reduced plant growth.	[86]
Expected environmental concentrations:						
CNTs (EU averages)						
surface water: 0.23 ng/L						
WWTP effluent: 4.0 ng/L						
WWTP biosludge: 0.15 mg/kg [86]						
Carbon black						
WWTP effluent: as low as 3.28–287.5 µg/L in London and as high as 5.91-673 µg/L in New York						
WWTP biosludge: as low as 530–2250 mg/kg in Shanghai and as high as 1220–5240 mg/kg in New York [88]						
**Silver-based NMs**						
Ag	Cucumber	7 day foliar exposure	NA	4 mg/plant	• Increased MDA contents; visible leaf yellowing	[93]
Ag (2 nm)	Tomato	2 weeks root exposure (uninoculated with mycorrhizal fungi) in soil	NA	12 mg/kg	• Decreased shoot dry weight (12–36 mg/kg)	[18]
Ag (2 nm)	Tomato	2 weeks root exposure (inoculated with mycorrhizal fungi) in soil	12 mg/kg	24 mg/kg	• Decreased shoot dry weight (24–36 mg/kg)	[18]
Ag (15 nm)	Tomato	2 weeks root exposure (uninoculated with mycorrhizal fungi) in soil	12 mg/kg	24 mg/kg	• Decreased shoot dry weight (24–36 mg/kg)	[18]
Ag (15 nm)	Tomato	2 weeks root exposure (inoculated with mycorrhizal fungi) in soil	36 mg/kg	NA		[18]
Ag with PEG coating	Tomato	56–62 days root exposure in soil	NA	10 mg/kg	• Reduced fruit yield and chlorophyll contents; increased oxidative stress parameters	[33]
Ag	Peanut	98 days root exposure in soil	NA	50 mg/kg	• Reduced growth and yield; increased antioxidant enzyme activities	[31]
Ag	Wheat	4 month root exposure in soil	NA	20 mg/kg	• Reduced growth	[32]
Expected environmental concentrations (EU averages):						
Sewage treatment effluent: 1–104 ng//L						
Surface (fresh) water: 0.03–3 ng/L						
Sludge-treated soils (100% degradation after one year): 20–1661 ng/kg						
Sludge-treated soils (100% persistence): 464–24,995 ng/kg [85]						
**Cerium-based NMs**						
CeO_2_	Bean	15 day root exposure in soil	NA	250 mg/kg	• Reduced total chlorophyll and proline contents	[94]
CeO_2_	Bean	15 day foliar exposure	NA	250 mg/plant	• Reduced anthocyanin, POD, and proline contents; reduced stomatal density	[94]
CeO_2_	wheat	90 days root exposure in soil	NA	125 mg/kg exposure in 1st and 2nd generations	• Reduced grain nutrient quality	[34]
CeO_2_	Soybean	3 weeks root exposure in soil	100 mg/kg	500 mg/kg	• Reduced photosynthesis rate	[24]
PVP- CeO_2_	Soybean	3 weeks root exposure in soil	100 mg/kg	500 mg/kg	• Reduced photosynthesis rate	[24]
Expected environmental concentrations (EU averages):						
Sewage treatment effluent: 20–889 ng/L						
Sludge-treated soils (100% degradation after one year): 528–19,012 ng/kg						
Sludge-treated soils (100% persistence): 11,212–560,423 ng/kg [85]						
**Copper-based NMs**						
CuO (aged)	Wheat	2 weeks root exposure in soil	NA	500 mg/kg	• Shorter root length	[27]
CuO (aged)	Lettuce	70 days root exposure in soil	NA	400 mg/kg	• Decreased biomass	[54]
CuO (unaged)	Wheat	2 weeks root exposure in soil	500 mg/kg	NA		[27]
CuO (unaged)	Lettuce	70 days root exposure in soil	NA	400 mg/kg	• Decreased biomass	[54]
CuO	Rice	Up to 88 days root exposure in soil	100 mg/kg	500 mg/kg	• Decreased growth and yield	[36]
CuO	Bell pepper	90 days root exposure in soil	250 mg/kg	500 mg/kg	• Reduced Zn contents in fruits and leaves	[37]
CuO	Cabbage	Up to 15 days foliar exposure	10 mg/plant	250 mg/plant	• Decreased gas and water exchange from blocked stomata and reduced dry weight	[9]
CuO	Lettuce	Up to 15 days foliar exposure	10 mg/plant	250 mg/plant	• Decreased gas and water exchange from blocked stomata and reduced dry weight	[9]
CuO	Peanut	145 days seed and root exposure in soil	NA	50 mg/kg	• Decreased total amino acid contents and altered fatty acid profile in peanut grains	[46]
Cu(OH)_2_	Spinach	7 days foliar exposure	1.8 mg/plant	18 mg/plant	• Reduced contents of antioxidant compounds and amino acids	[38,89]
Cu(OH)_2_	Corn	7 days foliar exposure	10 mg/plant	100 mg/plant	• Reduced leaf biomass and photosynthetic pigments	[89]
Cu(OH)_2_	Cucumber	7 days foliar exposure	25 mg/plant	NA		[95]
Expected environmental concentrations:						
WWTP effluent: Cu + CuO_x_: as low as >0.001–0.02 µg/L in London and as high as >0.001–0.03 µg/L in New York and Shanghai						
WWTP biosolids: Cu + CuO_x_: as low as >0.0–0.12 mg/kg in Shanghai to as high as 0.01–0.24 mg/kg in New York [88]						
**Titanium-based NMs**						
TiO_2_	Rice	130 days root exposure in soil (low CO_2_ conditions)	200 mg/kg	NA		[39]
TiO_2_	Rice	130 days root exposure in soil (high CO_2_ conditions)	NA	50 mg/kg	• Decreased plant biomass and yield	[39]
TiO_2_	Peanut	145 days seed and root exposure in soil	50 mg/kg	500 mg/kg	• Decreased total amino acid contents in peanut grains	[46]
TiO_2_	Rice	Full life-cycle root exposure in soil	750 mg/kg	NA		[40]
Expected environmental concentrations (EU averages):						
WWTP effluent: 16 μg/L						
WWTP sludge: 170 mg/kg [86]						
**Zinc-based NMs**						
ZnO	Bean	Up to 90 days root exposure in acidic soil	NA	3 mg/kg	• Decreased chlorophyll b and protein contents and altered antioxidant enzyme activity levels in leaves (increased GPOD activity at 15 and 30 days)	[14]
ZnO	Bean	Up to 90 days root exposure in calcareous soil	NA	3mg/kg	• Increased GPOD activity at 15 days	[14]
ZnO	Tomato	Up to 90 days root exposure in acidic soil	NA	3 mg/kg	• Increased MDA (indicative of lipid peroxidation) at 90 days. Decreased GPOD and increased CAT activities at 15 days. Plants died at ≥100 mg/kg	[14]
ZnO	Tomato	Up to 90 days root exposure in calcareous soil	NA	3 mg/kg	• Increased CAT activity at 15 and 30 days.	[14]
ZnO	Wheat	Full life-cycle exposure in fresh soil	6 mg/kg	NA		[41]
ZnO	Wheat	Full life-cycle exposure in weathered soil	5.98 mg/kg	NA		[41]
ZnO	Sorghum	Exposure time not provided. Foliar exposure	100 mL equivalent amount of Zn applied to roots	NA		[42]
ZnO	Sorghum	Exposure time not provided. Root exposure in soil	6 mg/kg	NA		[42]
ZnO	Maize	9 weeks root exposure in soil without organic P or AMF	500 mg/kg	NA		[43]
ZnO	Maize	9 weeks root exposure in soil with organic P, but without AMF	500 mg/kg	NA		[43]
ZnO	Maize	9 weeks root exposure in soil without organic P, but with AMF	500 mg/kg	NA		[43]
ZnO	Maize	9 weeks root exposure in soil with both organic P and AMF	500 mg/kg	NA		[43]
ZnO	Zucchini	21 days root exposure in vermiculate with dispersion	500 mg/L	NA		[22]
ZnO	Fenugreek (no inoculation with *Rhizobium melliloti*)	60 days root exposure in sand medium	NA	125 mg/kg	• Decreased nodule biomass	[19]
ZnO	Fenugreek (inoculated with *Rhizobium melliloti*)	60 days root exposure in sand medium	125 mg/kg	250 mg/kg	• Decreased nodule biomass	[19]
ZnO (bare)	Bean	>45 days root exposure in soil for S1 plants; unexposed S2 plants analyzed	500 mg/kg	NA	• Reduced Ni content in bean grains	[44]
ZnO (hydrophobically-coated)	Bean	>45 days root exposure in soil for S1 plants; unexposed S2 plants analyzed	NA	125 mg/kg		[44]
ZnO (bare)	Bean	>45 days root exposure until maturity in natural soil	NA	125 mg/kg		[26]
ZnO (hydrophobically-coated)	Bean	>45 days root exposure until maturity in natural soil	500 mg/kg	NA		[26]
ZnO (bare)	Bean	>45 days root exposure until maturity in organic-matter enriched soil	500 mg/kg	NA		[26]
ZnO (hydrophobically-coated)	Bean	>45 days root exposure until maturity in organic-matter enriched soil	500 mg/kg	NA		[26]
Expected environmental concentrations (EU averages):						
WWTP effluent: 2.3 μg/L						
WWTP sludge: 24 mg/kg [86]						

AMF = arbuscular mycorrhizal fungi; MDA = malondialdehyde; rGO = reduced graphene oxide; WWTP = waste water treatment plant.

## Supplementary Materials

The following are available online at https://www.mdpi.com/2079-4991/9/8/1094/s1, Section S1: Carbon-based NM interactions with plants, Section S2: Silver-based NM interactions with plants, Section S3: Copper-based NM interactions with plants, Section S4: Cerium-based NM interactions with plants, Section S5: Titantium-based NM interactions with plants, and Section S6: Zinc-based NM interactions with plants.
