# Supplementary material for: Nanomaterials in Plants: A Review of Hazard and Applications in the Agri-Food Sector
Source: Nanomaterials (Basel). 2019 Jul 30;9(8):1094. doi: 10.3390/nano9081094 (PMC6723683; doi:10.3390/nano9081094)
Supplement: Supplementary file 1 [file nanomaterials-09-01094-s001.pdf]

Supplementary Materials

Nanomaterials in Plants: A Review of Hazard and Applications in the Agri-Food Sector

Eva Kranjc * and Damjana Drobne

Department of Biology, Biotechnical Faculty, University of Ljubljana, Večna pot 111, 1000 Ljubljana, Slovenia
* Correspondence: eva.kranjc@bf.uni-lj.si

(Number of pages: 7; Number of figures: 0; Number of tables: 0)

S1. Carbon-based NM interactions with plants

Carbon-based NMs have been shown to exert positive effects on plant yield and nutrient contents under full life-cycle exposure in soil. They may enhance plant transpiration and nutrient bioavailability due to high surface porosity and a high density of active exchange sites. Sprouted gram seeds exposed to carbon nano-onions (CNOs; 10-30 μg/mL) before transplantation to soil showed significant, concentration-dependent increases in the size, weight, protein, electrolyte, and micronutrient contents (Mo, Cu, Mn, Ni, Zn, and Fe) of seeds produced at maturity compared to the control, without evidence of CNO translocation [1]. Full life-cycle exposure of two wheat cultivars to nanochitin (0.002-0.02 g/kg soil) resulted in increased uptake of N and K from soil and improved photosynthetic parameters, leading to increases in grain yield, and grain protein, Fe, and Zn contents [2].

S2. Silver-based NM interactions with plants

Silver NMs, which are most frequently used as antimicrobial agents in medical dressings and clothing items, have generally been found to exert negative effects on agricultural yield and nutritional value. Full life-cycle exposure of peanut [3] and wheat [4] to Ag NMs in soil resulted in dose-dependent increases in grain Ag content, decreased yield (87% and 91% decreased peanut yield at 500 and 2,000 mg/kg exposures, respectively; 16% and 24% decreased wheat yield at 200 and 2,000 mg/kg exposures, respectively), and decreased grain nutritional quality (12% increase in unhealthy saturated fatty acids and 3% decrease in healthy unsaturated fatty acids in peanuts at 500 mg/kg; 50% decreased Fe 15 2,000 mg/kg and 16-60% decreased Zn and Cu contents and protein contents at 200 and 2,000 mg/kg). In peanut plants, adverse changes were accompanied by significant, concentration dependent alterations in stress enzyme activities [3]. Similar results were shown for full life-cycle exposure of tomato to polyethylene glycol (PEG)-coated Ag NMs in soil, including significant translocation of Ag to tomato fruits, reduced plant uptake of N, P, and K macronutrients, and decreased fruit yield. Alterations to photosynthetic parameters were also measured along with increased oxidative stress parameters [5].

S3. Copper-based NM interactions with plants

As Cu is an essential plant micronutrient, a hormetic effect is frequently observed in which toxicity at excessive doses is preceded by physiological improvements at lower doses. For example, lettuce and cabbage foliar exposure to CuO NMs improved lettuce dry weight at a low dose (10 mg/plant), but decreased the dry weight, net photosynthesis, and stomatal conductance of both plants at a high dose (250 mg/plant), likely due to physical blockage of the stomata by CuO NM aggregates [6]. Among less beneficial outcomes, foliar exposure to a commercial $Cu(OH)_2$ spray (18 mg/plant) significantly decreased the contents of antioxidant compounds and amino acids in spinach [7], while soil exposure to CuO NMs (50 and 500 mg/kg soil) significantly decreased the 1,000-grain weight and total amino acid contents (33.6% and 21.1% for 50 and 500 mg/kg exposures, respectively) of peanut grains [8]. Full life-cycle rice exposure to CuO NMs significantly reduced

grain yield (500 and 1,000 mg/kg soil), but significantly increased grain Cu, Zn, and Fe contents (500 mg/kg) [9]. At the same exposure concentration in soil (500 mg/kg), full life-cycle exposure to CuO NMs did not significantly affect bell pepper agronomic parameters, however, fruit and leaf Zn levels were significantly decreased by 47% and 55%, respectively [10].

S4. Cerium-based NM interactions with plants

Cerium is not an essential element, however, it exhibits antioxidant enzyme-mimetic properties which can enhance plant growth [11]. Under drought conditions, foliar-applied CeO_2 NMs (2 mg/plant) elevated photosynthetic rates and lowered lipid peroxidation in sorghum plants, leading to a 31% increased seed yield relative to unwatered controls [12]. In a multi-generational study, first generation wheat plants exposed to CeO_2 NMs in soil (500 mg/kg) produced grains with increased biomass, amino acids, and fatty acids, which led to improved growth parameters in unexposed, second-generation plants. However, consecutive first- and second-generation exposures (125 mg CeO_2 NMs/kg) decreased grain nutrient contents (8, 8, 17, 20, and 19% decreases of Mn, Ca, K, Mg, and P respectively relative to second generation exposure alone) and minimized growth parameter increases that were measured from first generation exposure alone (500 mg CeO_2 NMs/kg in both generations). No evidence of CeO_2 NM translocation to grains was found, however, the mechanisms by which they affect multi-generational plant responses remain unclear [13].

S5. Titanium-based NM interactions with plants

TiO_2 is frequently incorporated into consumer products (e.g., sunscreens, paints, cements, and food products, such as chewing gums) and therefore has a high potential to accumulate in outdoor environments. Rice grown to maturity in TiO_2 NM-amended soil at high exposure concentrations (50-200 mg/kg) under either ambient or high CO_2 conditions showed significantly reduced grain yield (21% and 44% for 50 and 200 mg/kg exposures, respectively) and grain fat, protein, and total sugar contents under high CO_2 conditions (200 mg/kg), even though high CO_2 alone significantly increased grain yield in control plants relative to control plants grown under ambient CO_2 conditions. At 200 mg/kg TiO_2 NM-amended soil (high CO_2), rice grains contained significantly increased Ti, P, Mg, Ca, Mn, and Zn contents, which were likely due to the decreased size of the rice grains in this treatment group relative to the control groups [14]. Peanut plants root-exposed to TiO_2 NMs in soil produced grains with a significantly decreased 1,000-grain weight (50 and 500 mg/kg) and with decreased total amino acid contents (20.4%; 500 mg/kg) [8]. Conversely, full life-cycle rice exposure to TiO_2 NMs (25-750 mg/kg) in P-deficient soil significantly increased P uptake (5.8% increase in phytoavailable P at 750 mg/kg), and amino acids, palmitic acid, and glycerol contents in grains, without evidence of NM translocation [15].

S6. Zinc-based NM interactions with plants

As with Cu, Zn NMs can be expected to improve agronomic yield and nutrient values at appropriate concentrations due to its micronutrient status. Foliar application of a ZnO NM, B_2O_3 NM, and CuO NM mixture to soybean plants decreased the effects of drought conditions, significantly increasing chlorophyll contents, grain yield (36%), and grain N (35%), K (32%), Zn (68%), B (56%), and Cu (13%) relative to the control after full life-cycle exposure.[16] In response to ZnO NM exposure, bean and sorghum plants produced increased seed yield with increased nutrient contents regardless of whether the soil was fresh or used [17] or contained high or low NPK contents [18]. In another study, ZnO bioavailability and benefit to bean plants were significantly affected by soil type; those grown in organic matter-enriched soil (ES) had significantly increased seed yield (155%), which contained significantly higher concentrations of Zn (38%), K (64%), S (44%), P (83%), Mg (86%), Ca (70%), Fe (89%), and Mn (85%) relative to plants grown in natural soil (NS). The more beneficial outcomes obtained from plants grown in ES was attributed to a more neutral pH (more suitable for bean growth), the presence of organic matter (which increases metal bioavailability), and a higher concentration of bioavailable P [19].

References

1. Tripathi, K.M.; Bhati, A.; Singh, A.; Sonker, A.K.; Sarkar, S.; Sonkar, S.K. Sustainable Changes in the Contents of Metallic Micronutrients in First Generation Gram Seeds Imposed by Carbon Nano-onions: Life Cycle Seed to Seed Study. *ACS Sustain. Chem. Eng.* **2017**, *5*, 2906–2916.
2. Xue, W.; Han, Y.; Tan, J.; Wang, Y.; Wang, G.; Wang, H. Effects of Nanochitin on the Enhancement of the Grain Yield and Quality of Winter Wheat. *J. Agric. Food Chem.* **2017**, *66*, 6637–6645.
3. Rui, M.; Ma, C.; Tang, X.; Yang, J.; Jiang, F.; Pan, Y.; Xiang, Z.; Hao, Y.; Rui, Y.; Cao, W.; et al. Phytotoxicity of Silver Nanoparticles to Peanut (*Arachis hypogaea* L.): Physiological Responses and Food Safety. ACS Sustain. *Chem. Eng.* **2017**, *5*, 6557–6567.
4. Yang, J.; Jiang, F.; Ma, C.; Rui, Y.; Rui, M.; Adeel, M.; Cao, W.; Xing, B. Alteration of Crop Yield and Quality of Wheat upon Exposure to Silver Nanoparticles in a Life Cycle Study. *J. Agric. Food Chem.* **2018**, *66*, 2589–2597.
5. Das, P.; Barua, S.; Sarkar, S.; Chatterjee, S.K.; Mukherjee, S.; Goswami, L.; Das, S.; Bhattacharya, S.; Karak, N.; Bhattacharya, S.S. Mechanism of toxicity and transformation of silver nanoparticles: Inclusive assessment in earthworm-microbe-soil-plant system. *Geoderma* **2018**, *314*, 73–84.
6. Xiong, T.; Dumat, C.; Dappe, V.; Vezin, H.; Schreck, E.; Shahid, M.; Pierart, A.; Sobanska, S. Copper Oxide Nanoparticle Foliar Uptake, Phytotoxicity, and Consequences for Sustainable Urban Agriculture. *Environ. Sci. Technol.* **2017**, *51*, 5242–5251.
7. Zhao, L.; Huang, Y.; Adeleye, A.S.; Keller, A.A. Metabolomics Reveals $Cu(OH)_2$ Nanopesticide-Activated Anti-oxidative Pathways and Decreased Beneficial Antioxidants in Spinach Leaves. *Environ. Sci. Technol.* **2017**, *51*, 10184–10194.
8. Rui, M.; Ma, C.; White, J.C.; Hao, Y.; Wang, Y.; Tang, X.; Yang, J.; Jiang, F.; Ali, A.; Rui, Y.; et al. Metal oxide nanoparticles alter peanut (*Arachis hypogaea* L.) physiological response and reduce nutritional quality: a life cycle study. *Environ. Sci. Nano* **2018,** *5*, 2088–2102.
9. Peng, C.; Xu, C.; Liu, Q.; Sun, L.; Luo, Y.; Shi, J. Fate and Transformation of CuO Nanoparticles in the Soil–Rice System during the Life Cycle of Rice Plants. *Environ. Sci. Technol.* **2017**, *51*, 4907–4917.
10. Rawat, S.; Pullagurala, V.L.R.; Hernandez-Molina, M.; Sun, Y.; Niu, G.; Hernandez-Viezcas, J.A.; Peralta-Videa, J.R.; Gardea-Torresdey, J.L. Impacts of copper oxide nanoparticles on bell pepper (*Capsicum annum* L.) plants: a full life cycle study. *Environ. Sci. Nano* **2018**, *5*, 83–95.
11. Giese, B.; Klaessig, F.; Park, B.; Kaegi, R.; Steinfeldt, M.; Wigger, H.; Gleich, A.; Gottschalk, F. Risks, Release and Concentrations of Engineered Nanomaterial in the Environment. *Sci. Rep.* **2018**, *8*, 1565.
12. Djanaguiraman, M.; Nair, R.; Giraldo, J.P.; Prasad, P.V.V. Cerium Oxide Nanoparticles Decrease Drought-Induced Oxidative Damage in Sorghum Leading to Higher Photosynthesis and Grain Yield. *ACS Omega* **2018**, *3*, 14406–14416.
13. Rico, C.M.; Johnson, M.G.; Marcus, M.A.; Andersen, C.P. Intergenerational responses of wheat (*Triticum aestivum* L.) to cerium oxide nanoparticles exposure. *Environ. Sci. Nano* **2017**, *4*, 700–711.
14. Du, W.; Tan, W.; Peralta-Videa, J.R.; Gardea-Torresdey, J.L.; Ji, R.; Yin, Y.; Guo, H. Interaction of metal oxide nanoparticles with higher terrestrial plants: Physiological and biochemical aspects. *Plant Physiol. Biochem.* **2017**, *110*, 210–225.
15. Zahra, Z.; Waseem, N.; Zahra, R.; Lee, H.; Badshah, M.A.; Mehmood, A.; Choi, H.-K.; Arshad, M. Growth and Metabolic Responses of Rice (*Oryza sativa* L.) Cultivated in Phosphorus-Deficient Soil Amended with TiO_2 Nanoparticles. *J. Agric. Food Chem.* **2017**, *65*, 5598–5606.
16. Dimkpa, C.O.; Bindraban, P.S.; Fugice, J.; Agyin-Birikorang, S.; Singh, U.; Hellums, D. Composite micronutrient nanoparticles and salts decrease drought stress in soybean. *Agron. Sustain. Dev.* **2017**, *37*, 5.
17. Dimkpa, C.O.; Singh, U.; Bindraban, P.S.; Elmer, W.H.; Gardea-Torresdey, J.L.; White, J.C. Exposure to Weathered and Fresh Nanoparticle and Ionic Zn in Soil Promotes Grain Yield and Modulates Nutrient Acquisition in Wheat (*Triticum aestivum* L.). *J. Agric. Food* Chem. **2018**, *66*, 9645–9656.
18. Dimkpa, C.O.; White, J.C.; Elmer, W.H.; Gardea-Torresdey, J. Nanoparticle and Ionic Zn Promote Nutrient Loading of Sorghum Grain under Low NPK Fertilization. *J. Agric. Food Chem.* **2017**, *65*, 8552–8559.
19. Medina-Velo, I.A.; Dominguez, O.E.; Ochoa, L.; Barrios, A.C.; Hernández-Viezcas, J.A.; White, J.C.; Peralta-Videa, J.R.; Gardea-Torresdey, J.L. Nutritional quality of bean seeds harvested from plants grown

in different soils amended with coated and uncoated zinc oxide nanomaterials. *Environ. Sci. Nano* **2017**, *4*, 2336–2347.
